# Crystal structure of subunit VPS25 of the endosomal trafficking complex ESCRT-II

**DOI:** 10.1186/1472-6807-4-10

**Published:** 2004-12-04

**Authors:** Amy K Wernimont, Winfried Weissenhorn

**Affiliations:** 1 European Molecular Biology Laboratory (EMBL), 6 rue Jules Horowitz, 38042 Grenoble, France

## Abstract

**Background:**

Down-regulation of plasma membrane receptors via the endocytic pathway involves their monoubiquitylation, transport to endosomal membranes and eventual sorting into multi vesicular bodies (MVB) destined for lysosomal degradation. Successive assemblies of *E*ndosomal *S*orting *C*omplexes *R*equired for *T*ransport (ESCRT-I, -II and III) largely mediate sorting of plasma membrane receptors at endosomal membranes, the formation of multivesicular bodies and their release into the endosomal lumen. In addition, the human ESCRT-II has been shown to form a complex with RNA polymerase II elongation factor ELL in order to exert transcriptional control activity.

**Results:**

Here we report the crystal structure of Vps25 at 3.1 Å resolution. Vps25 crystallizes in a dimeric form and each monomer is composed of two winged helix domains arranged in tandem. Structural comparisons detect no conformational changes between unliganded Vps25 and Vps25 within the ESCRT-II complex composed of two Vps25 copies and one copy each of Vps22 and Vps36 [1,2].

**Conclusions:**

Our structural analyses present a framework for studying Vps25 interactions with ESCRT-I and ESCRT-III partners. Winged helix domain containing proteins have been implicated in nucleic acid binding and it remains to be determined whether Vps25 has a similar activity which might play a role in the proposed transcriptional control exerted by Vps25 and/or the whole ESCRT-II complex.

## Background

Endosomal compartments receive membrane bound cargo from both the biosynthetic and the endocytic pathways. Receptor downregulation by endocytosis includes transport to early endosomes and either recycling or sorting into late endosomes. The latter have the morphological characteristics of multivesicular bodies (MVB) [3] that can undergo homotypic fusion or heterotypic fusion with lysosomes, which deliver MVB cargo for proteolytic degradation [4]. In addition to receptor downregulation, MVB formation has been implicated in antigen presentation [5] and in the release of enveloped viruses [6,7].

Gene deletion and inactivation studies in yeast have identified 17 proteins that directly affect MVB formation (yeast class E compartment) by resulting in aberrant endosomal/vacuolar morphology [4]. All proteins are required for *vacuolar protein sorting *(VPS) into the class E compartment and are recruited to endosomal membranes from the cytosol in order to assemble into three ESCRT (***E****ndosomal ****S****orting ****C****omplexes ****R****equited for ****T****ransport*) complexes that function in MVB formation [8-11]. Receptor mono-ubiquitinylation has been shown to serve as a signal to enter the MVB pathway [12]. Initial recognition of ubiquitinated cargo by Vps27 recruits the ubiquitin binding protein Vps23 [11,13], which in turn leads to the assembly of the multi-protein complex ESCRT-I (VPS23, VPS28, and VPS37) [10]. ESCRT-I subsequently recruits ESCRT-II, composed of Vps22, Vps25, and Vps36, which in turn activates ESCRT-III subcomplexes [8,9]. Assembly of ESCRT-III at the endosome initiates the sorting and concentration of ubiquitinated cargo; ubiquitin is removed and Vps4, an AAA-type ATPase, dissociates the ESCRT complexes concomitantly with membrane invagination and budding of vesicles into the lumen of the endosome [4].

Two recent crystal structures of a core of the ESCRT-II complex reveal a trilobal complex, containing two copies of Vps25, one copy of Vps22 and the C-terminal region of Vps36. Each subunit is composed of two winged helix domains and an N-terminal region of Vps25 interacts with Vps22 and Vps36 [1,2].

Although ESCRT-II is essential for the MVB pathway, since cells missing ESCRT-II components fail to localize ESCRT-III to late endosomes [8,9] the complex has also been found "moonlighting" in the nucleus. The human and rat homologues of ESCRT-II were originally identified as the EAP complex (*ELL Associating Protein*; Vps22/EAP30; Vps25/EAP20; Vps36/EAP45), associated with the RNA polymerase II elongation factor ELL in the nucleus [14,15]. Consistent with a role in transcriptional control, yeast Vps22 (or SNF8) as well as Vps25 and Vps36 have been implicated in glucose-dependent gene expression control [15,16]. To date, it is not clear whether the role of ESCRT-II in MVB formation is independent of its function as a transcriptional activator or whether both processes are linked. Here, we report the crystal structure of full-length yeast Vps25, composed of two homologous winged-helix domains.

## Results and discussion

### Structure of Vps25

**Figure 1:**
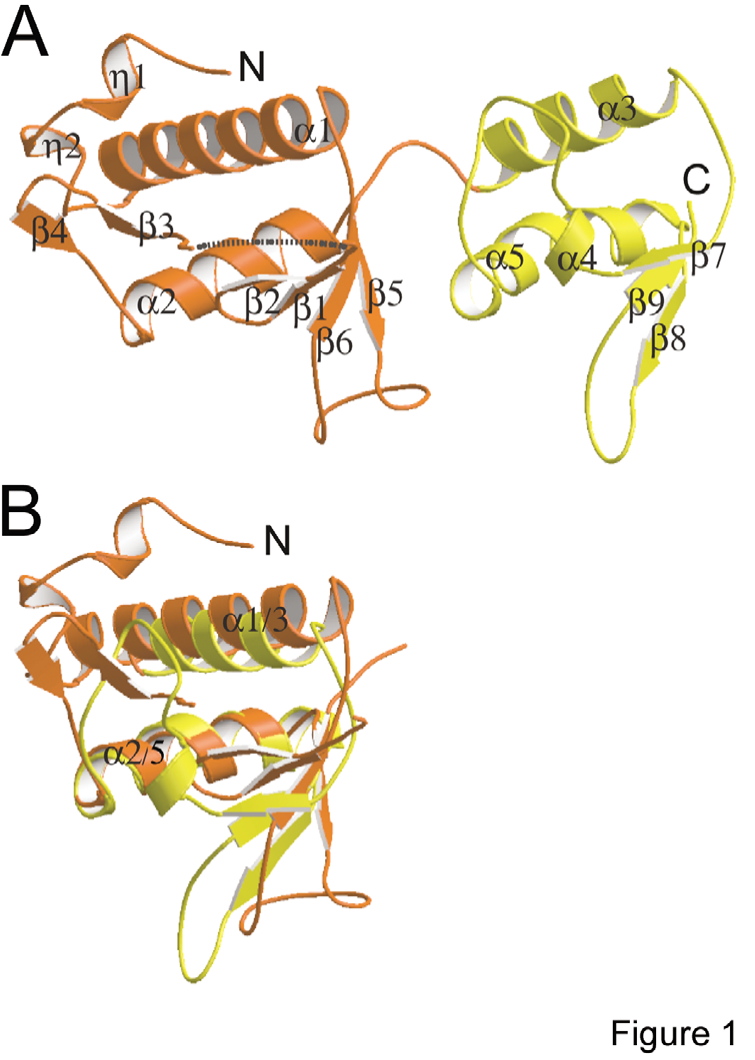
**Vps25 contains two winged helix domains arranged in tandem. **(A) Ribbon diagram of Vps25; the two domains are shown in orange and yellow. Secondary structure elements are labeled. The major missing loop region connecting strands 1 and 3 is indicated by a dashed line. (B) Superposition of the Calpha positions of the N- and C-terminal domains (residues 23 to 48 and 85 to 101 with corresponding C-terminal domain residues; r.m.s.d. 3.4 Å). Note that the positions of helices 1/3 and helices 2/5 as well as wing positions W1 match up well.

**Figure 2:**
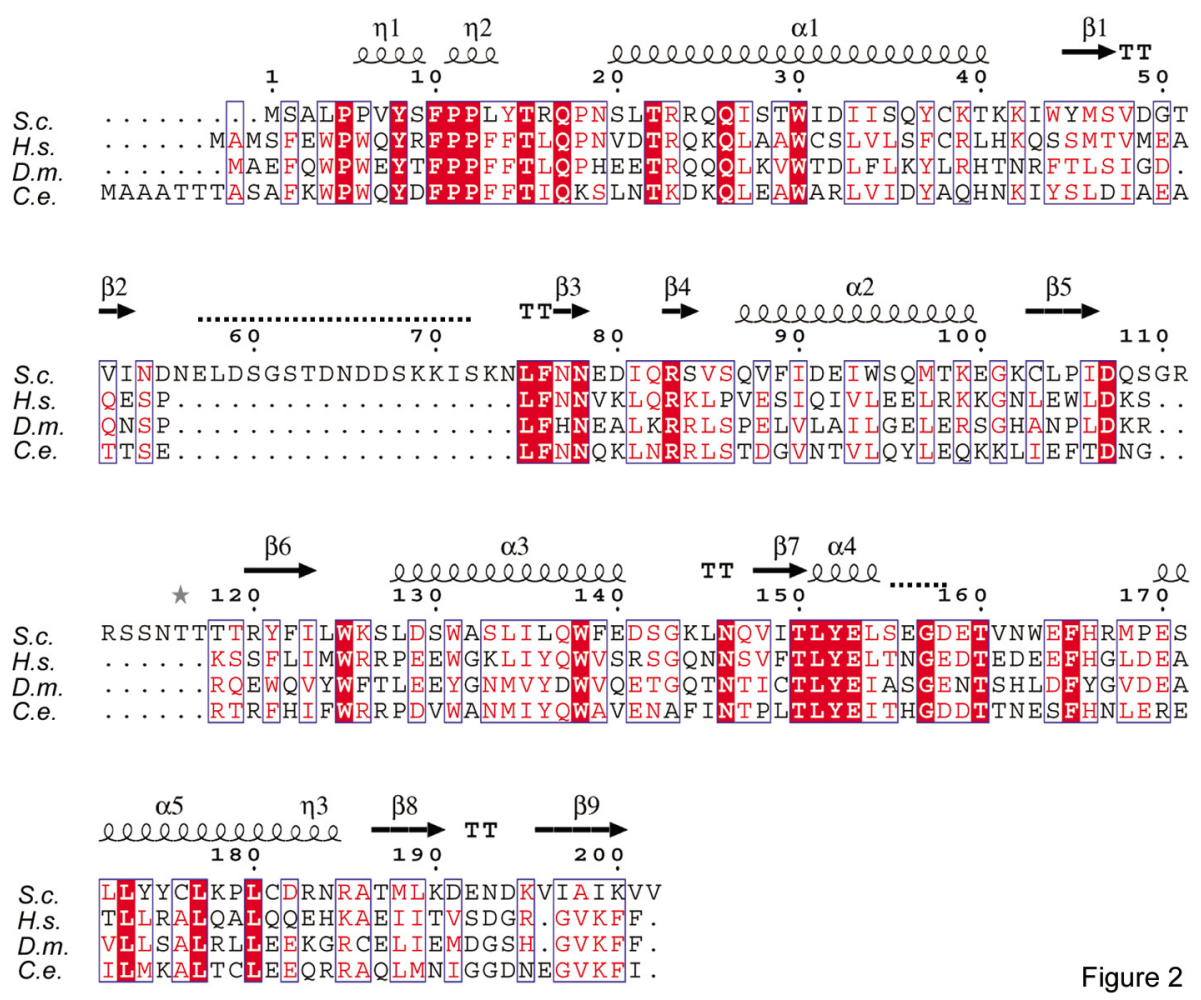
**Structure based sequence alignment of Vps25. **Sequences aligned using *S. cerevisiae *Vps25 (gene bank #CAA89632) and Vps25 orthologues from *H. sapiens *(#BE386260), *D. melanogaster *(#AAF59066) and from *C. elegans *(#T26073). Identical residues are shown on red background, similar residues are drawn in red and sequence similarity is underlined by blue boxes. Secondary structure elements are shown. Disordered regions in the Vps25 structure are indicated by dashed lines.

The structure of Vps25 was solved by single wavelength anomalous diffraction (SAD) using selenomethionine-derivatized crystals. Vps25 consists of two homologous winged helix domains as detected by the program GRATH  that are arranged in tandem (Figure 1A). Winged helix folds are compact alpha/beta structures with secondary structure elements arranged in a typical order (H1-S1-H2-H3-S2-W1-S3-*W2optional*) [17], which fold into a mostly helical part followed by a twisted anti-parallel beta-sheet and two large loops (wings, W). The fold of Vps25 deviates slightly from the canonical fold. The N-terminal domain 1 (residues 1 to 126) contains two additional N-terminal 3/10 helices, implicated in the interaction with either Vps22 or Vps36 [1,2], followed by the canonical helix 1 and strand 1. It lacks canonical helix 2, which instead folds into a large disordered loop followed by strands 3 and 4 that connects to helix 2 (at the corresponding position of canonical helix 3). Strands 5 and 6 then form, together with strand 1, a twisted anti-parallel beta-sheet with wing W1 protruding from the structure (Figure 1A and Figure 2). Domain 1 also lacks wing W2, as in the cases of winged helix domain containing transcription factors E2F4 and DP2 [18]. Strand 6 flows directly into domain 2, which also has a canonical winged helix fold except for the absence of wing W2 (Figure 1A and Figure 2). Domains 1 and 2 are tightly packed against each other and their C alpha atoms can be superimposed with an r.m.s. deviation of 3.4 Å (Figure 1B), confirming their structural relatedness. The domain interface is dominated by van der Waals contacts including conserved and non conserved residues Trp44, Phe122, Leu104, Leu124, Trp125 in domain 1 and Leu128, Trp131, Met168, Pro169 and Leu172 in domain 2 (Figure 2).

### Structural comparision of unliganded Vps25 and Vps25 in complex with Vps22 and Vps36 (ESCRT-II)

**Figure 3:**
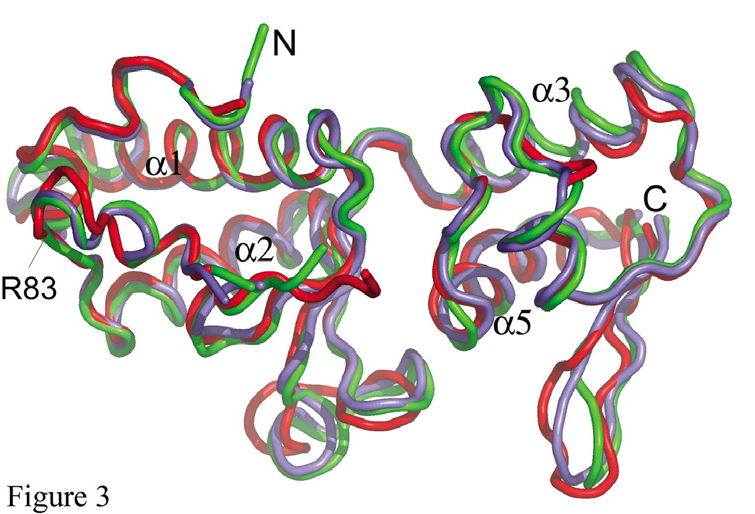
**Comparison of unliganded and liganded Vps25. **Superposition of unliganded Vps25 (red) with Vps25 from both ESCRT-II structures [1, 2] (blue, pdb code 1U5T chain C; green, pdb code 1W7P chain B). The peptide backbones are shown as coils. Vps25 is shown in the same orientation as in figure 1A. The position of Arg83 is indicated by an arrow.

Two recent crystal structures of the ESCRT-II core reveal trilobal structures with head to tail interactions of one copy of Vps25 with Vps22 and the other copy of Vps25 with Vps36 at the center. In both cases a conserved proline rich N-terminal region of Vps25 (Figure 2) together with conserved Arg83 mediate key interactions [1,2]. Therefore it was of interest to analyse whether Vps25 undergoes any conformational changes upon participation in ESCRT-II complex formation. Superposition of the C alpha atoms with one copy of Vps25 from either ESCRT-II complex structure ([1,2]; pdb entries 1U5T and 1W7P) revealed r. m. s. displacements of 1.2/1.2 Å (residues 3 to 51), 1.5/1.7 Å (residues 74 to 155) and 2.3/2.9 Å (residues 159–199) respectively. The major changes are confined to both wings W1 and W2 indicating their conformational flexibility (Figure 3). In contrast, the conserved N-terminal segment, which is implicated in Vps22 and Vps36 interactions shows no substantial changes (Figure 3).

In the unliganded Vps25 structure, this helical segment constitutes the 1192 Å^2 ^dimerization interface of two identical Vps25 dimers present in the asymmetric crystal unit. The dimer contact is mediated by hydrophobic residues Pro5, Pro6, Val7, Phe10, Pro11, and Pro12, which is similar to the contact region described for Vps25 interactions with Vps22 and Vps36 [1,2]. In the Vps25 structure Arg83 does not participate in dimerization but hydrogen bonds to Thr15 instead of forming salt bridges with either Vps36 Asp548 or Vps22 Asp214 as observed in the ESCRT-II complex [1,2]. Arg83 locates to a beta hairpin (strand 4; Figure 2) in the unliganded form of Vps25. Although the position of Arg83 is unchanged in all Vps25 structures (Figure 3) the position of the preceding loop region varies which might be due to differences in secondary structure assignment [1,2]. Therefore Vps25 seems to dock as a rigid body onto either Vps22 or Vps36 upon ESCRT-II complex formation. Although we do not detect Vps25 dimer formation *in vitro*, a dimeric form of Vps25 might be stabilized through other unknown interactions.

### Structural homology of Vps25 with nucleic acid binding winged helix domains

Analysis of the full-length structure with DALI [19] revealed seven structural homologues displaying nucleic acid binding winged helix domains with a Z score above 5 for Vps25 domain 1. The top two hits were the selenocysteine-specific elongation factor fragment (PDB 1lva, Z score 6) and double-stranded RNA specific adenosine deaminase (ADAR) Z-alpha domain (PDB 1qbj, Z score 5.5). Winged helix family members interact with nucleic acids mostly via the "specificity helix" that binds to the major groove of the DNA with two flanking loops contributing to DNA interactions [17]. Superposition of Vps25 domain 1 onto the winged helix domain of E2F-4 bound to DNA [18] matching the "specificity helices" (Vps25 helix H2) revealed a potential fit with only minor clashes at the helix H1 loop region (data not shown). A potential nucleic acid interaction of Vps25 might be interesting in light of the described role of Vps25 and the other ESCRT-II subunits in glucose-dependent gene regulation [15,16] and complex formation with RNA polymerase II elongation factor ELL [14,15], although no biochemical data exist so far to support such a proposed function.

### Vps25 participates in protein complex formation

**Figure 4:**
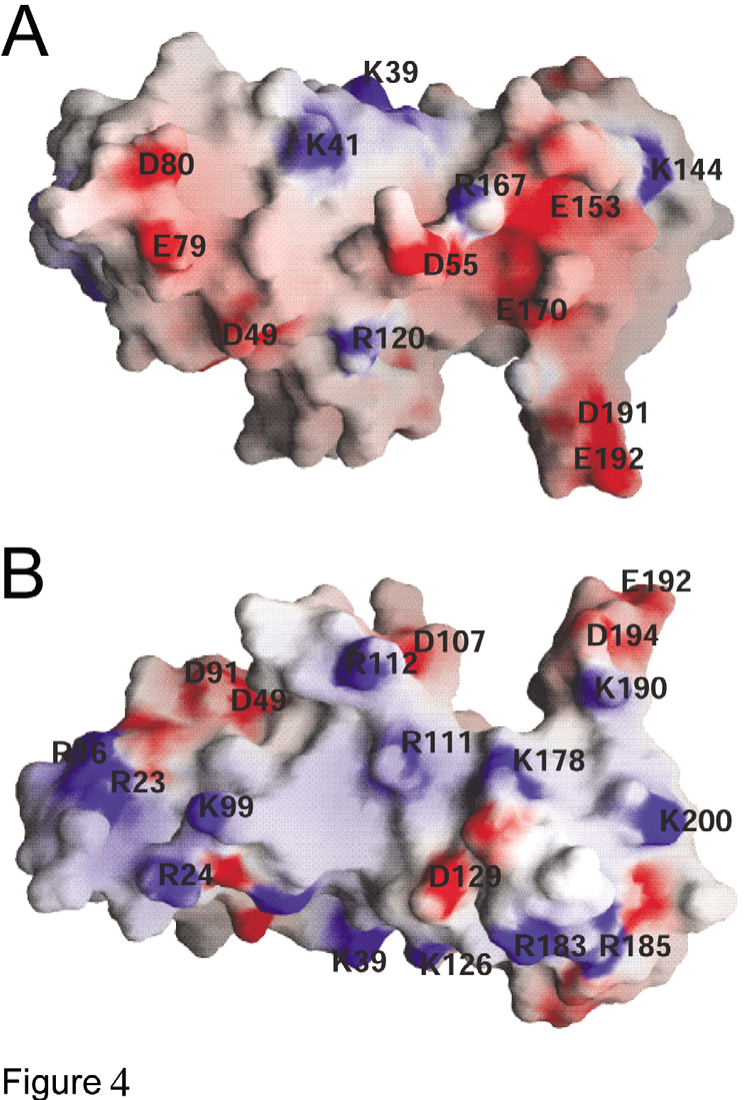
**Surface charge distribution of Vps25. **(A) Surface potential representation of Vps25 with regions where electrostatic potential <-10 k_B_T are red, while those >+10 k_B_T are blue (k_B_, Boltzmann constant; T, absolute temperature). (B) Horizontal rotation (180°). Exposed residues are labeled for orientation. Note that one face of the molecule carries a mainly negative charge (A) while the other one carries a mainly positive charge (B).

The ESCRT-II complex assembles at the endosomal membrane downstream of ESCRT-I and recruits ESCRT-III subcomplexes [8-10]. Consistent with such a sequential assembly, further ESCRT-II interactions of Vps25 have been described, namely with Vps28 (ESCRT-I) and with Vps20 (CHMP6; ESCRT-III) [7,20]. Surface electrostatic potential maps of Vps25 reveal a negatively charged surface within domain 2 that is characterized by a patch of conserved residues such as Glu153, Glu170 and Tyr152 (Figure 4A and Figure 2). Tyr152 is also part of the highly conserved domain 2, helix 4 (Figure 2). Domain 2 is the outer domain of Vps25 in the ESCRT-II complex and this region would thus be freely accessible for potential interaction(s) with Vps28 or Vps20. Similarly, basic residues (Lys99 and Arg23) potentially implicated in nucleic acid recognition are part of a conserved patch on domain 1 (Figures 4B and 2).

Vps25 contains additional features, which are unique to *S. cerevisiae*, as evidenced from multiple sequence analysis [15,16]. Vps25 orthologues have a shorter strand 2 to strand 3 connection (19 residues), whose sequence is composed of mostly charged residues and is disordered in our structure as well as in the ESCRT-II structures [1,2]. Furthermore, domain 1 wing W1 is shorter (7 residues) (Figure 2), which might indicate *S. cerevisiae *unique protein-protein interaction sites.

## Conclusions

Clear evidence suggests that ESCRT-II recruitment is involved in MVB formation leading to plasma membrane receptor downregulation [4]. On the other hand ESCRT-II seems to play a role in transcription regulation [15]. Similarly, other ESCRT components such as Tsg101 (***T****umor ****s****usceptibility ****g****ene*; Vps23; ESCRT-I) and members of the CHMP protein family (ESCRT-III; ***Ch****romatin ****M****odifying ****P****rotein*; ***Ch****arged ****M****ultivesicular body ****P****rotein*) are also found to act in the nucleus as well as in the cytosol and at endosomal membranes [21-23]. Interestingly, both Vps25 and Vps36 have been implicated in regulating stress and pheromone response pathways [24] and pheromone receptor *Ste2 *is downregulated via the endosomal pathway [12]. Similarly, SNF8 (Vps 22; EAP30), Vps36 and Vps25 are all directly involved in derepression of glucose-repressed genes, which might be linked to sorting of sucrose receptors via the endosomal pathway [15,25]. Protein sorting into MVB involves monoubiquitylation of cargo, which is recognized by ESCRT members. ESCRT-II Vps36 contains an ubiquitin binding NZF zinc finger motif that is necessary for protein sorting [26]. Therefore, ESCRT-II complexes may sense the turnover of specific ubiquitylated receptors at the endosomal membrane together with other unknown signals. As ESCRT-II only transiently associates with endosomal membranes [9] a signal within the MVB process might induce nuclear localization of ESCRT-II, where it could stimulate gene expression leading to up or down regulation of specific membrane receptors.

## Methods

### Protein expression, purification and crystallization

Full length yeast Vps25 DNA (gene bank #CAA89632) was cloned into expression vector pETM30 (EMBL, Protein Expression Facility) and the Vps25 GST fusion protein was expressed in *E. coli *BL21 codon+ cells. For purification, cell pellets from 6 liter cultures were lysed in 150 mls of buffer A (50 mM Tris-HCl, pH 8.5, 200 mM NaCl, 0.2 mM DNaseI, 2 mM β-ME, 2 complete EDTA-free protease inhibitor tablets (Pierce)) and 0.1 mg/ml lysozyme for one hour on ice. The cell lysate was cleared by centrifugation and loaded onto a GST-sepharose (Pharmacia) column. The column was extensively washed with buffer B (50 mM Tris pH 8.5, 200 mM NaCl) and Vps25 fusion protein was eluted with buffer B containing 5 mM reduced glutathione. GST was then removed by TEV cleavage (w/w; 1:200) at 4°C overnight. His-tagged GST and TEV were subsequently both removed on a Ni^2+ ^chelating sepharose column. Vps25 was further purified on a superdex75 column (Pharmacia) in buffer C (50 mM Tris 8.5, 200 mM NaCl, 2 mM βME). Selenomethione-labeled Vps25 was produced using standard procedures and purified as described above.

Crystallization conditions for Vps25 (7 mg/ml) were first determined by screening 600 conditions using a Cartesian crystallization robot. Initial conditions were refined using the hanging drop method, and the final crystallization condition (100 mM Na cacodylate pH 6.5, 200 mM Mg or Ca acetate, 5–7% glycerol, and 15–18% polyethylene glycol 8000) produced rectangular- and wedge-shaped selenomethionine-labeled Vps25 crystals in the same drop. Native Vps25 crystallized initially only with rectangular morphology and wedge-shaped crystals were produced by microseeding with the original SeMet crystals. For cryogenic data collection, the crystals were equilibrated in 25% glycerol and flash cooled in a gaseous nitrogen stream at 100 K.

Crystallization produced rectangular crystals that belong to space group P422 with unit cell dimensions a = b = 78 Å, c = 54 Å and diffract to 3.2 Å resolution. However, all data sets collected from these crystals proved to be almost perfectly merohedrally twinned. The second crystal form, wedge-shaped, belonged to space group P2_1_2_1_2_1 _with unit cell dimensions as indicated (table 1), contained 4 molecules per asymmetric unit, diffracted X-rays to 3.1 Å resolution and was used for structure solution.

**Table 1:** Data Collection and Refinement.

Crystal	VPS25-SEMET		VPS25-NATIVE	
Space Group	P2_1_2_1_2_1_		P2_1_2_1_2_1_	
Wavelength	0.97914		0.931	
Unit Cell (Å)				
a	53.44		53.36	
b	124.11		123.66	
c	139.48		140.30	
Resolution (outer shell) (Å)	100-3.20 (3.31-3.20)		100 - 3.10 (3.21-3.10)	
Total Reflections (outer shell)	111407 (10016)		65477 (6064)	
Unique Reflections	29230		16330	
Completeness (%) (outer shell)	98.9 (93.1)		93.1 (91.3)	
Rmerge (outer shell)	0.090 (0.335)		0.053 (0.307)	
Average I/sigma (outer shell)	12.3 (4.9)		20.9 (4.9)	
**Phasing**				
Number of Se Sites	14			
SOLVE FOM	0.351			
RESOLVE FOM (ncs)	0.694			
**Refinement**				
Resolution (outer shell) (Å)	25.0-3.10 (3.29-3.10)			
Number of reflections (test set)	16404 (790)			
R factor	0.275			
Free R factor	0.327			
Number of protein/solvent atoms	5760/16			
Average B factor (Å^2^)	51.3			
Rms deviation bond lengths (Å)	0.009			
Ramachandron	Mol A	Mol B	MolC	MolD
Most favored Additionally favored	83.8 15.7	79.5 20.5	82.1 17.9	67.2 32.8

### Data Collection

Native data for Vps25 were collected at the European Synchrotron Radiation Facility (ESRF) beamline ID14-EH3 and data from SeMet-labeled crystals were collected at the ESRF beam line ID29 at three wavelengths (table 1). Data were processed and scaled with XDS [27].

### Phasing and refinement

Significant radiation damage had occurred for data collected at the inflection and remote wavelengths, therefore only data collected at the peak wavelength (table 1) were used for SAD phasing. ShelXD [28] was used to find 14 out of 16 selenium sites, which were further refined with SOLVE [29]. Four-fold non-crystallographic symmetry was imposed on the sites in addition to solvent flattening with RESOLVE [30]. Phasing statistics are listed in table 1. The initial model was built with O [31] guided by the SeMet positions and clear tryptophan (7 per mol) and tyrosine (8 per mol) densities followed by refinement with CNS [32]. Strict four-fold NCS and phases were initially kept throughout the initial chain-tracing and refinement. During model building it was observed that molecules A and B and molecules C and D are arranged in the same dimer configuration and strict NCS was changed to restrained NCS during refinement. The packing also indicated tight interactions between molecules A, B, and C while molecule D showed only very few crystal contacts yet formed the "bridge" between two-dimensional layers formed by molecules A, B and C. The electron density maps for molecules A, B, and C were clear and well defined, while electron density for molecule D was poorly defined for side chains and loops. The model was improved by alternating cycles of model building and conjugate gradient minimization and restrained individual B-factor refinement using CNS [32]. The final coordinates were refined against the native dataset (30 to 3.1 Å) using the MLHL maximum likelihood target with the RESOLVE phases as constraint and retaining the original test set reflections. In the final stage of refinement, a maximum likelihood target and model phases alone were used.

The final model lacks two to five flexible loops (molecule mol A, residues 56–72, 114–115, 156–157; mol B, residues 53–73, 157–158; mol C, residues 57–72, 155–158; mol D, residues 19–21, 55–73, 107–120, 156–160, 185–186). Accordingly, mol D is poorly defined (43 residues missing out of 204). The final R factor and R free (0.275/0.327) reflect missing residues and the poor model for molecule D. The model exhibits otherwise overall good stereochemistry with no outliers in the Ramachandran plot as defined in PROCHECK (table 1) [33]. The coordinates have been deposited in the RCSB Protein Data Bank accession code 1XB4 [PDB:1XB4].

### Structure analysis

Figures were generated using coordinates of molecule C with programs MOLSCRIPT [34], Raster 3D [35], ESPript [36], GRASP [37] and PyMOL . Sequences were aligned using Clustalx [38]. Secondary structure elements were assigned using the program DSSP [39]. The buried surface was calculated with CNS [32] and the program LSQMAN was used for superpositioning of C-alpha positions [40].

## Authors' contributions

W.W. conceived of the study, and participated in its design, coordination and writing of the manuscript. W.W. expressed, purified and established initial crystallization conditions and participated in data collection. A.K.W. carried out data collection, structure solution and refinement and participated in writing of the manuscript. All authors read and approved the final manuscript.
